# Antecedents and consequences of academic help-seeking in online STEM learning

**DOI:** 10.3389/fpsyg.2024.1438299

**Published:** 2024-11-06

**Authors:** Sungjun Won, Yujin Chang

**Affiliations:** ^1^Department of Education, Gongju National University of Education, Gongju, Republic of Korea; ^2^Department of Education, Chungbuk National University, Cheongju, Republic of Korea

**Keywords:** academic help-seeking, online learning, sense of belonging, environmental fixed mindset, STEM education

## Abstract

**Introduction:**

College students often encounter challenges or ambiguity in online learning, which they cannot overcome independently, and therefore, require help. However, relatively little is known about how academic help-seeking can be supported in online contexts and about its potential benefits. The present study investigated the role of academic help-seeking in online STEM learning and its contextual antecedents.

**Methods:**

A total of 213 college students, enrolled in an introductory Engineering course, completed an online survey. Their survey responses and academic record data were analyzed.

**Results:**

Results of path analysis indicated that adaptive help-seeking was positively related to retention intention, whereas expedient help-seeking was negatively related to the choice of future courses. In addition, avoidant help-seeking was negatively related to retention intention and major declaration status and positively to disorganized studying. Results also showed that sense of belonging and environmental fixed mindset served as significant predictors of academic help-seeking.

**Discussion:**

Findings indicate that academic help-seeking is related to successful online STEM learning. Therefore, fostering online learning contexts in which students perceive more sense of belonging and less environmental fixed mindset is crucial.

## Introduction

Despite the significant contribution of Science, Technology, Engineering, and Mathematics (STEM) to our daily lives, the dropout rate in STEM education has been considerably high for the past several decades (Glass et al., 2013; Seymour and Hewitt, 1997; van den Hurk et al., 2019). Numerous studies have attempted to identify factors influencing students’ persistence and academic performance in STEM fields. However, most of these studies have focused on face-to-face learning, and surprisingly little is known about STEM learning in online contexts. At least two reasons suggest the necessity of investigating online STEM learning. First, online learning has increasingly become an important component of higher education, particularly during the coronavirus disease 2019 (COVID-19) pandemic. Second, online learning differs from face-to-face learning in that it requires greater degrees of self-regulation with increased autonomy, as the less structured nature of online environments demands that students independently manage their learning activities, such as goal-setting, time management, and seeking assistance (Broadbent and Poon, 2015; Dabbagh and Kitsantas, 2004). Therefore, it is essential to understand how STEM students learn in online environments.

The present study focuses on academic help-seeking, a critical self-regulated learning strategy (Karabenick and Berger, 2013; Karabenick and Gonida, 2018). Specifically, we postulate that academic help-seeking could play a pivotal role in online STEM learning. Considering the repeatedly reported difficulty of STEM learning itself (Koenig et al., 2012; Seymour and Hewitt, 1997), seeking assistance in their learning seems critical for students. Notably, academic help-seeking is a socially mediated strategy involving other social figures. One key difference between online and face-to-face learning is the presence of an instructor and other students and how they interact and communicate (Broadbent and Lodge, 2021; Kitsantas and Chow, 2007). Although prior work has well documented the importance of academic help-seeking in face-to-face settings (e.g., Karabenick, 2003, 2004), this difference makes it difficult to draw any solid conclusions and further calls for research evaluating its importance in online settings. We thus investigate the utility of academic help-seeking in predicting students’ choice, retention intentions, and performance in online STEM learning.

This study also examines sense of belonging and environmental entity theory as two antecedents of academic help-seeking and educational outcomes in online STEM learning. Sense of belonging has been linked to students’ retention and achievement (e.g., Hausmann et al., 2009; Lewis and Hodges, 2015). Environmental entity theory (i.e., environmental fixed mindset), recently introduced by researchers (Good et al., 2012) and particularly pervasive in STEM fields (Lytle and Shin, 2020), has also been found to be associated with students’ retention intentions and achievement in STEM fields (Good et al., 2012). Students’ perceptions of their instructors and peers may promote or hinder their decision to ask for help. Hence, the present study investigates if sense of belonging and environmental fixed mindset could predict students’ choices, retention intentions, and performance in online STEM learning directly and indirectly via academic help-seeking.

## Literature review

### Academic help-seeking

Given the increased complexity of learning materials in postsecondary education, students inevitably face insurmountable challenges or ambiguity, which can interfere with their optimal learning. To seek help and assistance in such circumstances, students should be able to monitor their understanding, recognize their needs for seeking help, and translate this recognition into action (Karabenick and Berger, 2013). In other words, academic help-seeking consists of students’ cognition, motivation, and behavior regulation. Accordingly, it has been consistently conceived of as a self-regulated learning strategy, which enables students to perform a more active and constructive role in their learning (Karabenick, 2003; Karabenick and Berger, 2013; Karabenick and Dembo, 2011; Karabenick and Gonida, 2018; Newman, 2000; Ryan et al., 2001). Indeed, there is compelling evidence that academic help-seeking is conducive to academic success (Fong et al., 2023). Specifically, college students’ *adaptive help-seeking*, asking for hints or explanations necessary for learning and task mastery, has been linked to greater cognitive and behavioral engagement, less anxiety, and better academic performance (e.g., Karabenick, 2003, 2004; Karabenick and Knapp, 1991; Kitsantas and Chow, 2007; Micari and Calkins, 2021).

However, it should be noted that students do not always ask for help to improve their knowledge and skills (Butler, 1998; Karabenick, 2003, 2004; Newman, 2000; Ryan et al., 2005). A theoretical distinction has been drawn between adaptive help-seeking and expedient help-seeking as suggested by Nelson-Le Gall (1985). Unlike adaptive help-seeking, which can ultimately facilitate learning, in *expedient help-seeking*, students attempt to minimize their efforts in completing their academic tasks by simply asking for solutions to problems without explanation or requesting others to perform the tasks. Prior research has provided empirical evidence indicating that expedient help-seeking is detrimental to academic success (Fong et al., 2023). Specifically, college students’ expedient help-seeking was positively linked to anxiety and negatively linked to academic performance (Karabenick, 2003). Similar findings have been consistently reported in prior work investigating adolescent populations (e.g., Cheong et al., 2004; Ryan et al., 2005; Ryan and Shim, 2012).

Furthermore, although college students are generally able to monitor their learning processes and recognize gaps in their skills or knowledge, some students are reluctant to ask for much-needed help from their instructors and peers (Collins and Sims, 2006; Karabenick and Knapp, 1991). Asking for help may be regarded as a sign of personal inadequacy and overdependence, leading to embarrassment and loss of self-esteem (Nelson-Le Gall, 1985). More importantly, it has been claimed that by avoiding help-seeking when needed, students place themselves in a disadvantageous position for academic success (Ryan et al., 2001). Consistent with this claim, prior research documented that college students’ *avoidant help-seeking* was related to their lower exam performance and higher anxiety (Karabenick, 2003). Similarly, Karabenick (2004) showed that college students’ avoidant help-seeking was negatively related to academic performance. These findings are broadly consistent with those from previous research on adolescent students. Adolescent students’ avoidant help-seeking has been negatively associated with test scores and grade point average and positively with anxiety (Ryan et al., 2005; Ryan and Shin, 2011).

### Academic help-seeking in online learning

Academic help-seeking has been primarily investigated in traditional, face-to-face learning contexts. The role of help-seeking in online learning has not been extensively investigated, thus, representing a notable gap in the literature. Compared to face-to-face learning, students are generally expected to plan, organize, and control their learning much more under their own direction. Furthermore, students have fewer opportunities to interact with their instructors and peers directly. To ask for help from their instructors, students typically send emails or chat messages or post help requests on online forums (e.g., Cheng et al., 2013), which is different from academic help-seeking in face-to-face settings. Considering its different nature, it is critical to evaluate academic help-seeking and its importance for academic success in online learning settings.

As observed in face-to-face contexts (e.g., Karabenick, 2003, 2004), a few studies have demonstrated that students could benefit from academic help-seeking in online learning. For instance, academic help-seeking was positively associated with college students’ participation in online learning activities and test scores (Schworm and Gruber, 2012). Similarly, the combination of academic help-seeking and flipped learning intervention successfully increased college students’ self-efficacy and course involvement (Chyr et al., 2017). Additionally, Liu (2017) reported that preservice teachers’ online help-seeking was positively related to their engagement in self-regulated learning. These studies have provided informative insights, yet several notable shortcomings in the studies make it difficult to draw any firm conclusions concerning the role of academic help-seeking in online learning. Specifically, these studies tested academic help-seeking in the context of blended learning, which consists of both face-to-face and online learning (Chyr et al., 2017; Schworm and Gruber, 2012), and focused only on adaptive help-seeking (Chyr et al., 2017; Liu, 2017). Furthermore, one study manipulated both academic help-seeking and flipped learning simultaneously, making it impossible to evaluate the effect of academic help-seeking only (Chyr et al., 2017). These shortcomings along with the paucity of studies point to a need for research examining the role of academic help-seeking in online learning contexts.

Furthermore, due to the COVID-19 pandemic, a rapid transition to online learning was required, presenting numerous challenges to students (Hensley et al., 2022). It became more difficult to understand learning materials and complete tasks, making it necessary for students to seek assistance or additional explanations for learning and task mastery. Simply looking for solutions to problems without fully understanding them or avoiding help-seeking altogether could hinder their learning. Consequently, academic help-seeking could play a key role in helping students overcome these challenges and achieve their academic goals. Therefore, we investigated this among college students taking online courses during the pandemic, particularly those majoring in STEM fields.

### Academic help-seeking in STEM learning

Due to the considerably high attrition rates in STEM fields, understanding factors influencing students’ STEM-related choices has received significant attention (Lykkegaard and Ulriksen, 2019; Miller and Wai, 2015; van den Hurk et al., 2019). Various individual and contextual factors have been found to contribute to STEM dropout (e.g., Ball et al., 2017; Graham et al., 2013; Seymour and Hewitt, 1997; van den Hurk et al., 2019). Amidst such factors, academic difficulties, including inadequate preparation, language barriers, and the nature of conceptual difficulties, act as barriers keeping students from achieving STEM degrees. As such, academic help-seeking could likely promote STEM retention. By requesting assistance, students can avert possible failure, maintain engagement, successfully progress in their program, and ultimately attain a degree (Newman, 2002). Indeed, students’ engagement in self-regulated learning has been broadly linked to their retention in the program (e.g., Patterson et al., 2014; Peck et al., 2018; Reparaz et al., 2020).

Despite its potential importance in STEM fields, little attention has been paid to STEM college students’ academic help-seeking. A few studies that examined the association between help-seeking strategies and academic performance in math or science consistently documented the adaptive nature of academic help-seeking (Horowitz et al., 2013; Szu et al., 2011). Specifically, Horowitz et al. (2013) found that strategic help seekers performed better in chemistry than those who avoided help-seeking. Similarly, Szu et al. (2011) reported that higher achieving students demonstrated more engagement in help-seeking behaviors earlier in the semester than lower achieving students in organic chemistry. However, these studies have mostly focused on academic performance concerning academic help-seeking. Thus, the relation between students’ academic help-seeking and their choice or persistence in STEM fields remains unclear.

The present study investigated whether STEM students’ academic help-seeking strategies can be used to understand their choice and retention intentions in STEM fields. Specifically, we examined the role of adaptive, expedient, and avoidant help-seeking in predicting not only students’ academic performance but also their choice of courses and retention intentions in STEM fields. It should be noted that students often experience confusion and uncertainty in online learning. In addition to students’ course grades and major declaration status, we tested if academic help-seeking was related to students’ disorganization (i.e., perceptions of not knowing what to do or how to study for the course; Elliot et al., 1999) as another indicator of their academic performance.

### Sense of belonging and environmental fixed mindset

College students do not always utilize available resources and assistance provided by their postsecondary institutions or use them appropriately (Karabenick, 2003). Hence, instructors and administrators are particularly interested in encouraging students to fully utilize such resources and assistance. To address this issue, students’ academic help-seeking should be understood within the academic and social contexts in which they function, as suggested by models of self-regulated learning (Pintrich and Zusho, 2007; Zimmerman, 1989, 1995, 2000). In particular, academic help-seeking involves other social figures from whom students request and receive help and assistance (Newman, 2000). However, little is known about online learning contexts that can promote college students’ adaptive help-seeking and reduce their expedient and avoidant help-seeking. We focused on sense of belonging and environmental fixed mindset as two potential predictors of students’ adaptive, expedient, and avoidant help-seeking in online settings.

Sense of belonging refers to the extent to which students feel accepted by their instructors and peers and perceive themselves as a valuable part of their learning environment (Goodenow, 1993; Osterman, 2000). While sharing some commonalities with social support and positive relationships with instructors and peers, sense of belonging differs in that it reflects perceived social membership within the school community rather than individual relationships. It is likely that students’ sense of belonging plays a critical role in academic help-seeking for two reasons. First, sense of belonging is closely associated with social and emotional adjustment. Specifically, college students are more likely to be socially accepted and less likely to feel anxious or lonely when they perceive a strong sense of belonging (e.g., Gummadam et al., 2016; Ostrove and Long, 2007). With the reduced anxiety and increased security from being accepted by their community members, including instructors and peers, students would feel more comfortable asking for help. Second, sense of belonging facilitates students’ adoption of the ideals and values shared within their school or community, such as the importance and utility of learning (Goodenow, 1993; Goodenow and Grady, 1993). The internalization of the importance and utility of STEM learning, as shared within the STEM community, is likely to encourage STEM students’ academic help-seeking. Indeed, one recent study reported that college students’ sense of belonging was positively related to adaptive help-seeking, whereas it was not related to expedient help-seeking (Won et al., 2021). Although not specifically for academic help-seeking, several studies have also provided relevant evidence showing that sense of belonging is related to students’ engagement in self-regulated learning (Kennedy and Tuckman, 2013; Won et al., 2018). In sum, prior work has indicated that college students’ sense of belonging could inform if and how they seek help with their learning.

The present study also focused on students’ perceptions of environmental fixed mindset. Environmental fixed mindset refers to the extent to which students perceive an entity-oriented learning environment (Good et al., 2012). Unlike self-theories of intelligence that focus on students’ own beliefs about the fixedness or malleability of their ability or intelligence, environmental fixed mindset focuses on how students perceive people around them with regard to their views on intelligence or ability. It also differs from instructors’ fixed mindset in that it encompasses the fixed mindset of both instructors and peers, reflecting a broader perspective on the collective views of intelligence within the students’ environment. Previous research has shown that these perceptions of entity views in one’s environment can play a pivotal role in motivation and learning (Good et al., 2012; Rattan et al., 2012). We postulated that students’ perceptions of how the STEM community views intelligence or ability would serve as another critical contextual factor predicting their help-seeking in online STEM learning.

Although the role of environmental fixed mindset has not yet been examined in predicting academic help-seeking, it can be inferred from previous work on individual-level fixed mindset (i.e., the self-theories of intelligence). Prior studies have consistently reported that when students hold an entity view of intelligence (i.e., the belief that intelligence is a fixed trait, and they cannot do much to change it), they are less likely to seek adaptive forms of help and more likely to engage in help-avoidance behaviors (Mihlon, 2010; Shih, 2007).

Indeed, given the inherently social nature of academic help-seeking, it is likely that not only students’ own beliefs on intelligence but also their perceptions of how instructors and peers, whom they ask for needed assistance, view intelligence could promote or hinder their academic help-seeking. If students perceive that their community sees intelligence as a fixed trait, they may avoid asking questions or seeking help out of fear of appearing incompetent. In contrast, when students believe that their community members deem intelligence a malleable trait, they may be less worried about looking incompetent and more likely to engage in adaptive help-seeking. That is, when students believe their learning environment emphasizes growth and development, they are more likely to view help-seeking as a natural part of the learning process, fostering greater academic engagement and success. As noted in the literature on help-seeking, students’ competence concerns are among the primary factors contributing to help-seeking avoidance (Ryan et al., 2001). Indeed, it should also be noted that fixed views on intelligence or ability are particularly prevalent in STEM learning contexts (Lytle and Shin, 2020). Therefore, environmental fixed mindset is likely to play a pivotal role in explaining STEM students’ academic help-seeking.

Based on these findings, we investigated sense of belonging and environmental fixed mindset as two determinants of college students’ help-seeking in online STEM learning. Notably, sense of belonging and environmental fixed mindset have been linked to students’ academic performance and retention (e.g., Good et al., 2012; Hausmann et al., 2009; Lewis and Hodges, 2015). Thus, we further examined whether students’ academic help-seeking would serve as a pathway through which sense of belonging and environmental fixed mindset relate to choice, retention intentions, and academic performance.

### The present study

The primary goal of this study was to investigate the role of academic help-seeking in online STEM learning by addressing three research questions. First, to what extent does undergraduate STEM students’ academic help-seeking predict their choice, retention intentions, and performance (disorganization, course grades, major declaration status) in online STEM learning? Drawing on prior research investigating face-to-face learning (e.g., Karabenick, 2003; Kitsantas and Chow, 2007), we expected that adaptive help-seeking would positively predict students’ choice, retention intentions, and performance, whereas expedient and avoidant help-seeking would show null or negative relations.

Second, to what extent do sense of belonging and environmental fixed mindset predict academic help-seeking in online STEM learning? Consistent with existing evidence (e.g., Won et al., 2021), we hypothesized that students’ sense of belonging in their STEM community would positively predict their use of adaptive help-seeking and negatively predict their expedient and avoidant help-seeking. On the contrary, we expected that students’ perceptions of an entity-oriented STEM environment would negatively predict their adaptive help-seeking and positively predict their expedient and avoidant help-seeking.

Third, does academic help-seeking mediate the relations of sense of belonging and environmental fixed mindset with STEM choice, retention intentions, and performance? We hypothesized that students’ academic help-seeking would serve as pathways linking sense of belonging and environmental fixed mindset to students’ choice, retention intentions, and performance. Specifically, sense of belonging would be positively associated with choice, retention intentions, and performance via increased adaptive help-seeking and decreased expedient and avoidant help-seeking. The opposite pattern was expected with environmental fixed mindset.

## Method

### Participants and procedure

A total of 213 students (*M*_age_ = 19.0, *SD*_age_ = 1.96) were recruited from an introductory Engineering course (13 sections) at a large public university located in Western Canada. This course was fully offered online during the spring term of 2021 due to the COVID-19 pandemic and consisted of asynchronous lectures and synchronous labs. In general, students engaged with course material through asynchronous lectures, where they independently learned key concepts at their own pace. During the synchronous labs, students applied theoretical knowledge and completed exercises. Most undergraduate courses were also offered online during this semester. This was not the students’ first encounter with online learning environments, as many had experienced online coursework during previous terms as part of the adjustments to pandemic-related restrictions. Notably, this introductory course is mandatory for first-year Engineering students to declare specific Engineering programs (e.g., computer science and biomedical engineering). The majority of the students were male (70.9%) and in their first year at the university (83.1%).

An online survey was administered toward the end of the spring term of 2021, and academic record data were obtained after the semester ended. Specifically, the online survey assessed students’ sense of belonging, environmental fixed mindset, academic help-seeking, disorganization, and retention intentions, whereas their course grades and major declaration status were obtained from the academic records. The university’s Institutional Review Board approved the protocol for this study.

### Measures

All constructs were measured at a domain-specific level, and students were directed to respond to items specific to the domain of Engineering and the introductory Engineering course in which they were enrolled. The Cronbach’s αs obtained from the current study are reported in Table 1.

**Table 1:** Descriptive statistics for observed variables.

Variable	*M*	*SD*	Variance	Skewness	Kurtosis	Min. observed	Max. observed	*α*
Sense of belonging	4.07	1.07	1.14	−0.27	−0.07	1.00	6.00	0.93
Environmental fixed mindset	2.52	0.86	0.74	0.38	−0.07	1.00	5.00	0.88
Adaptive help-seeking	3.75	0.72	0.51	−0.72	0.81	1.00	5.00	0.80
Expedient help-seeking	2.12	0.77	0.59	0.74	0.37	1.00	5.00	0.82
Avoidant help-seeking	2.16	0.75	0.56	0.49	−0.40	1.00	4.33	0.80
Choice	5.42	1.13	1.27	−0.33	−0.74	2.00	7.00	0.66
Retention intentions	5.27	0.85	0.72	−1.36	1.48	1.83	6.00	0.83
Disorganization	3.96	1.62	2.61	−0.11	−0.92	1.00	7.00	0.93
Course grade	90.49	4.69	22.00	−0.34	1.23	72	100	–
Major declaration status	0.74	0.44	0.19	−1.08	−0.83	0	1	–

*N = 213. Min., minimum; Max., maximum.*

#### Sense of belonging

We adopted the sense of belonging scale from Bollen and Hoyle (1990). The scale consisted of three items assessing students’ sense of belonging to the Engineering community (e.g., “I see myself as a part of the Engineering community”). Students responded to the items using a 6-point Likert scale, ranging from 1 (*strongly disagree*) to 6 (*strongly agree*). Prior work using this scale has documented its good internal consistency and positive relation to college students’ persistence (e.g., Hausmann et al., 2007; Hurtado and Carter, 1997).

#### Environmental fixed mindset

We assessed environmental fixed mindset using the four-item scale derived from Good et al. (2012). This scale measured the extent to which students perceive people in engineering to have a fixed mindset (e.g., “People in Engineering believe that people have a certain amount of engineering intelligence, and they cannot really do much to change it”). Students responded to the items using a 5-point Likert scale, ranging from 1 (*strongly disagree*) to 5 (*strongly agree*). This scale has demonstrated acceptable reliability and its negative relation to students’ intentions to pursue math in prior research (Good et al., 2012; Rattan et al., 2012).

#### Academic help-seeking

Academic help-seeking was assessed using the Students’ Help-Seeking measure developed by Ryan and Shim (2012) and Ryan et al. (2009). A total of 18 items measured students’ *adaptive help-seeking* (six items; e.g., “If there is something I do not understand, I ask someone for help so I can learn it”), *expedient help-seeking* (six items; e.g., “If I do not understand something, I usually want someone to just give me the answer”), and *avoidant help-seeking* (six items; e.g., “If my coursework is too hard for me, I just do not do it rather than asking for help”) in the introductory Engineering course they were taking. The focus of this measure is primarily on seeking assistance related to learning, understanding course content and materials, and completing academic tasks. Students completed the measure using a 5-point Likert scale, ranging from 1 (*not at all true*) to 5 (*very true*). In previous research, this scale has shown acceptable reliability and theoretically consistent relations to students’ behavior and emotional engagement and academic performance (e.g., Shim et al., 2016).

#### Choice

Students’ choice of future Engineering courses was measured using the scale from Wolters (2004). This scale consisted of four items (e.g., “I look forward to taking more Engineering courses in the future”), and students responded to the items using a 7-point Likert scale, ranging from 1 (*strongly disagree*) to 7 (*strongly agree*). Responses to negatively worded items were reverse coded such that high values indicated higher intentions to take more Engineering courses in the future. Prior research using the scale has documented acceptable reliability and its positive relations to motivation and retention intentions (e.g., Wu et al., 2020).

#### Retention intentions

As an indicator of persistence, students’ retention intentions in their Engineering major were measured using the scale developed by Perez et al. (2014). Students responded to six items (e.g., “At the present time, I am likely to switch to a major that is not in an Engineering field”) using a 6-point Likert scale, ranging from 1 (*strongly disagree*) to 6 (*strongly agree*). Responses to negatively worded items were reverse coded such that higher values represented higher intentions to remain in the Engineering major. This scale has shown its good reliability and positive relations to academic achievement in prior research (e.g., Hilts et al., 2018).

#### Disorganization

We measured students’ perceived disorganization in the introductory Engineering course by adopting the five-item scale from Elliot et al. (1999). Students responded to the items (e.g., “I’m not sure how to study for this online course”) using a 7-point Likert scale, ranging from 1 (*not at all true of me*) to 7 (*very true of me*). The scale has been adopted in prior work and demonstrated good reliability and its positive relations to procrastination and avoidance intentions and negative relation to academic performance (e.g., Jiang et al., 2018).

#### Course grades

Students’ grades for the introductory Engineering course were obtained from the academic records. The course grades could range from 0 to 100.

#### Major declaration status

Students’ specific Engineering major declaration status was collected from the academic records. To qualify for the major declaration, students were required to complete 24 credits and achieve the minimum grade point average of C+ with no course grade less than C.

### Overview of analysis

Descriptive statistics and correlations were computed using SPSS 24. Then, path analysis was conducted to address our three research questions using Mplus 7.31 (Muthén and Muthén, 1998–2012). A diagonally weighted least square estimator (WLSMV) was utilized given that one of the outcomes, students’ major declaration status, was binary (Muthén and Muthén, 1998–2012). As such, probit regression coefficients were reported for the paths related to major declaration status, whereas linear regression coefficients were reported for all the other paths.

We specified a path model in which academic help-seeking was postulated as a pathway linking perceived academic contexts to educational outcomes. Given the predominance of male students, a common occurrence in STEM majors, we included gender as a covariate in the path model. Additionally, academic year was included as another covariate. Despite the introductory course being mandatory primarily for first-year students, it was also open to students in other academic years.

To test the indirect effects of perceived academic contexts on educational outcomes via academic help-seeking, a bootstrap procedure with 1,000 bootstrapping samples and 95% bias-corrected confidence intervals were utilized (MacKinnon et al., 2007; Williams and MacKinnon, 2008). Model fit was evaluated based on several fit indices. Besides the chi-square statistics (*χ*^2^), we used the Tucker–Lewis Index (TLI), Comparative Fit Index (CFI), and Root Mean Square Error of Approximation (RMSEA) based on the recommendation by Hu and Bentler (1999).

## Results

### Descriptive statistics and correlations

Descriptive statistics for all major variables are presented in Table 1. Most of the mean scores fell near the middle of the response scale, except for choice (*M* = 5.42) and retention intentions (*M* = 5.27), which were somewhat higher. The mean score for adaptive help-seeking (*M* = 3.75 on a 5-point scale) indicates a tendency for students to engage in effective help-seeking behaviors, slightly above the midpoint of the scale. In contrast, the mean scores for expedient (*M* = 2.12) and avoidant help-seeking (*M* = 2.16) suggest that students were relatively less engaged in these forms of help-seeking compared to adaptive help-seeking. Additionally, the mean score for sense of belonging (*M* = 4.07 on a 6-point scale) was above the midpoint, indicating a slightly positive sense of connection to the engineering community. The mean course grade was 90.5, and approximately 73.7% of students successfully declared their specific Engineering major at the end of the term. Skewness and kurtosis statistics indicated that all observed variables approximate a normal distribution. All scales showed acceptable degrees of internal consistency (0.80 ≤ *α* ≤ 0.93), except for the choice scale. The reliability was somewhat low (α = 0.66), and we suspected that it was due to the two reverse-worded items of the four-item scale. Missing rates ranged from 0 to 0.9%, which were minimal.

As presented in Table 2, sense of belonging was positively correlated with adaptive help-seeking (*r* = 0.16), whereas environmental fixed mindset was positively correlated with expedient help-seeking (*r* = 0.22). Adaptive help-seeking showed a positive correlation with retention intentions (*r* = 0.24). In contrast, expedient help-seeking was positively correlated with disorganization (*r* = 0.23) and negatively with retention intentions (*r* = −0.22). Similarly, avoidant help-seeking was positively correlated with disorganization (*r* = 0.36) and negatively with retention intentions (*r* = −0.35), course grade (*r* = −0.15), and major declaration status (*r* = −0.27).

**Table 2:** Bivariate correlations for observed variables.

Variable	1	2	3	4	5	6	7	8	9	10
1. Sense of belonging	–									
2. Environmental fixed mindset	0.07	–								
3. Adaptive help-seeking	0.16^*^	0.10	–							
4. Expedient help-seeking	−0.07	0.22^**^	0.08	–						
5. Avoidant help-seeking	−0.13	0.08	−0.28^***^	0.46^***^	–					
6. Choice	0.16^*^	−0.13	0.10	−0.29^***^	−0.24^***^	–				
7. Retention intentions	0.20^**^	−0.14^*^	0.24^***^	−0.22^**^	−0.35^***^	0.44^***^	–			
8. Disorganization	−0.12	0.10	0.01	0.23^***^	0.36^***^	−0.26^***^	−0.26^***^	–		
9. Course grade	0.09	0.02	0.08	−0.05	−0.15^*^	0.16^*^	0.16^*^	−0.18^*^	–	
10. Major declaration status	0.02	−0.05	−0.03	−0.11	−0.27^***^	0.03	0.22^**^	−0.23^***^	0.32^***^	–

*N* = 213. ^*^p < 0.05, ^**^p < 0.01, ^***^p < 0.001.

### Path model

**Figure 1:**
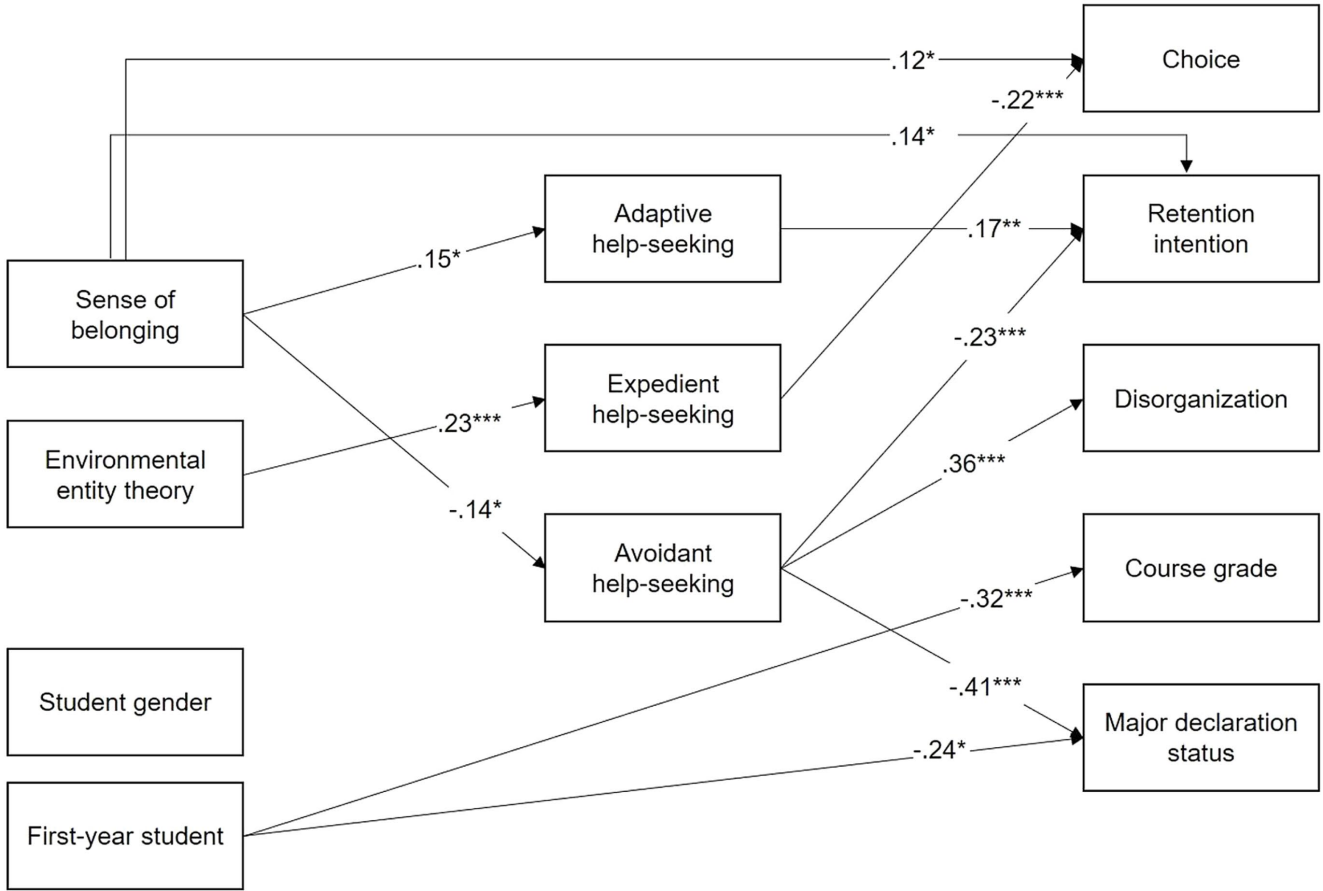
Standardized path coefficients from the path model. Only statistically significant paths at *p* < 0.05 are presented. Error terms and residual covariances are omitted for clarity. First-year student (others = 0, first-year student = 1). *p < 0.05, **p < 0.01, ***p < 0.001.

We examined a path model with students’ gender and academic year as covariates. The model fit the data well, χ^2^(6, *N* = 213) = 2.581, *p* = 0.859 (CFI = 1.000, TLI = 1.000, RMSEA = 0.000). As presented in Figure 1, students’ gender was not related to any of the outcomes, whereas students’ academic year significantly predicted their final course grades (*b* = −3.95, *β* = −0.32, *p* < 0.001) and major declaration status (*b* = −0.65, *β* = −0.24, *p* = 0.019). That is, first-year students were less likely to perform well in the introductory online Engineering course and declare specific Engineering majors.

As expected, sense of belonging was significant in predicting academic help-seeking. Specifically, sense of belonging positively predicted adaptive help-seeking (*b* = 0.10, *β* = 0.15, *p* = 0.021) and negatively predicted avoidant help-seeking (*b* = −0.10, *β* = −0.14, *p* = 0.027). However, sense of belonging was not significant in predicting expedient help-seeking. Additionally, sense of belonging was directly associated with retention intention (*b* = 0.11, *β* = 0.14, *p* = 0.013) and choice (*b* = 0.13, *β* = 0.12, *p* = 0.043). The opposite predictive pattern emerged with environmental fixed mindset. Environmental fixed mindset was predictive of expedient help-seeking (*b* = 0.20, *β* = 0.23, *p* < 0.001). However, environmental fixed mindset failed to predict adaptive or avoidant help-seeking and any indicators of choice, retention intentions, and performance.

Consistent with our hypotheses, adaptive help-seeking positively predicted retention intention (*b* = 0.20, *β* = 0.17, *p* = 0.003), whereas expedient help-seeking negatively predicted choice (*b* = −0.33, *β* = −0.22, *p* < 0.001). Avoidant help-seeking negatively predicted retention intention (*b* = −0.26, *β* = −0.23, *p* < 0.001) and major declaration status (*b* = −0.55, *β* = −0.41, *p* < 0.001) and positively predicted disorganization (*b* = 0.77, *β* = 0.36, *p* < 0.001). Neither adaptive, expedient, nor avoidant help-seeking, however, significantly predicted course grades.

### Indirect effects

Based on the path analysis results, we evaluated the indirect effects of sense of belonging and environmental fixed mindset via academic help-seeking. Table 3 presents all significant indirect effects based on a bootstrap procedure with 1,000 bootstrapping samples and 95% bias-corrected confidence intervals. In general, results support our hypotheses that students’ perceptions of social contexts were related to their choice, retention intentions, and academic performance not only directly but also indirectly via academic help-seeking.

**Table 3:** Estimates of indirect effects.

					95% bias-corrected bootstrapped CI	
			*β*	*b*	Lower 2.5%	Upper 2.5%
Sense of belonging	→ Adaptive help-seeking	→ Retention intentions	0.025	0.020	0.002	0.056
	→ Avoidant help-seeking	→ Retention intentions	0.033	0.026	0.002	0.068
		→ Disorganization	−0.050	−0.077	−0.184	−0.004
		→ Major declaration status	0.058	0.055	0.003	0.127
Environmental fixed mindset	→ Expedient help-seeking	→ Choice	−0.050	−0.065	−0.141	−0.022

Specifically, the indirect effects of sense of belonging on retention intention were significant via both adaptive help-seeking and avoidant help-seeking. In addition, the indirect effects of sense of belonging on disorganization and major declaration status were significant through avoidant help-seeking. Lastly, the indirect effect of environmental fixed mindset on choice was also significant via expedient help-seeking.

## Discussion

In the present study, we examined the antecedents and consequences of students’ academic help-seeking within the context of online STEM learning. The results revealed that college students’ academic help-seeking was related to their choice, retention intentions, and academic performance in STEM fields. Furthermore, we found that sense of belonging and environmental fixed mindset could be used to understand students’ academic help-seeking in online learning.

### Academic help-seeking in online STEM learning

Our results show that academic help-seeking relates to students’ choice and retention intentions. Specifically, students’ adaptive help-seeking was positively related to their retention intentions in STEM fields, and their avoidant help-seeking was negatively related to their intentions. That is, the more students reportedly asked for hints or explanations necessary for learning and task mastery or the less students avoided necessary help, the more they demonstrated their intentions to remain in STEM fields. Interestingly, adaptive help-seeking and avoidant help-seeking were not predictive of choice. In contrast, expedient help-seeking emerged as a significant predictor of students’ choice. Specifically, students who reportedly asked for solutions to problems without explanation or requested others to do their tasks were less likely to take more Engineering courses in the future. These findings indicate that students’ attempts to minimize their effort in learning or completing academic tasks could be further manifested in refraining from taking non-mandatory courses.

In prior work, the importance of academic help-seeking has been typically supported by its relations to academic performance (e.g., Karabenick, 2003; Micari and Calkins, 2021). Our findings extend prior work and provide insights into the role of academic help-seeking in promoting students’ choice and retention intentions, particularly within STEM fields. Due to the inherently difficult and conceptually complex nature of STEM courses (Koenig et al., 2012; Seymour and Hewitt, 1997), a substantial proportion of students drop out or switch to non-STEM majors (Glass et al., 2013; van den Hurk et al., 2019). This issue is further compounded in online environments, where limited instructor interaction and less structured coursework present additional challenges. Given the increasing shift toward online learning in higher education, our findings are particularly promising. Helping students adopt more effective approaches to these learning challenges could promote their choice of courses and retention intentions in STEM fields. Greater focus on the potential role of academic help-seeking in STEM retention is clearly one important direction for future research.

Our findings concerning the relations between students’ academic help-seeking and academic performance were somewhat mixed. We examined three different aspects of students’ academic performance, including disorganized studying, course grades, and major declaration status. On the one hand, the three types of academic help-seeking did not predict students’ course grades. This result stands in contrast to other studies in which academic help-seeking has been found to predict students’ course grades (e.g., Micari and Calkins, 2021). On the other hand, students’ avoidant help-seeking was positively related to their disorganized studying (i.e., not knowing how to study effectively and what to study for the online Engineering course) and negatively related to their major declaration status. Put differently, students who showed help-seeking avoidance were more likely to report difficulties in studying for the online course and less likely to meet the requirements (i.e., minimum course credits and cumulative GPA) to declare a specific Engineering major.

There are several possible reasons for the mixed pattern of results. First, help-seeking behavior may not have been necessary for success in a particular course. As noted in prior research, specific course characteristics (e.g., course difficulty, grading) could moderate the relations between help-seeking and academic performance (Credé and Phillips, 2011). Indeed, a meta-analysis study showed that compared to individual course grades, students’ use of learning strategies in general had stronger correlations with GPA, which can be deemed a more holistic representation of college students’ academic performance (Credé and Kuncel, 2008). Providing support for this speculation, academic help-seeking was significantly associated with students’ major declaration status, determined based on their cumulative GPA and course credits, in the present study. Second, as documented in several studies (Credé and Phillips, 2011; Roszkowski, 2013), there might be a curvilinear relationship between academic help-seeking and course grades. Specifically, very high- or low-achieving students are less likely to seek assistance in their learning (Credé and Phillips, 2011; Fong et al., 2023), which might not have been captured in our analysis. Third, the institution’s grading policies during the data collection could also have contributed to the mixed findings. Due to the pandemic and students’ increased academic stress, the institution switched to a more lenient grading system, which could have masked the differences in students’ academic performance. Relatedly, students’ avoidant help-seeking was positively related to their disorganized studying. That is, students who avoided seeking help reported more difficulties and ambiguity in their course learning, but the course grades based on the lenient grading system may not have accurately captured them.

Overall, this study provides empirical evidence supporting the pivotal role of academic help-seeking in online STEM learning. Specifically, the different types of academic help-seeking related to various aspects of educational outcomes, including students’ choice of future courses, retention intentions, disorganization, and major declaration status. Our findings support the conclusion that discouraging help-seeking avoidance could be as important as promoting adaptive help-seeking for students’ academic success and retention in STEM fields. To our knowledge, this is the first empirical study to evaluate the three different types of academic help-seeking and link them to students’ STEM choice and retention intentions in online learning settings. It is also noteworthy that the present study evaluated the importance of academic help-seeking by utilizing not only students’ self-reported responses but also their course grades and major declaration status obtained from academic records at the end of the semester. This use of objective data enhanced the rigor of the findings, providing a more reliable evaluation of academic help-seeking and its significance.

### Sense of belonging and environmental fixed mindset as predictors of academic help-seeking

Students’ sense of belonging was positively associated with their reported use of adaptive help-seeking. That is, when students perceived that they were an essential part of the Engineering community and were accepted and supported by the community members, they were more likely to ask for assistance or explanation that could ultimately promote their understanding of course material. This finding is consistent with one recent study showing the positive link between sense of belonging and adaptive help-seeking (Won et al., 2021) and, more broadly, prior work documenting the relations of teacher support and classroom peer climate (Ryan et al., 2005; Ryan and Shim, 2012; Shim et al., 2013), as well as instructor relatedness and peer relatedness (Oh et al., 2024), with adaptive help-seeking. In contrast, sense of belonging was negatively related to students’ avoidant help-seeking. The more students perceived sense of belonging in their Engineering community, the less they reportedly avoided asking for help when needed. This finding adds to prior work showing that students with higher levels of avoidant help-seeking reported lower levels of teachers’ academic and emotional support (Ryan et al., 2005) and lower peer relatedness (Oh et al., 2024), which is necessary for building sense of belonging (Allen et al., 2018).

In addition to sense of belonging, this study also found the significant role of environmental fixed mindset in predicting college students’ expedient help-seeking. When students perceived that their instructors and peers in the Engineering community view Engineering intelligence or ability as a fixed trait, they were more likely to ask for solutions to problems without explanation or ask others to do their tasks. This finding could be explained by the well-established associations between threat to self-esteem and help-seeking reported by prior research (Arbreton, 1993; Karabenick and Knapp, 1991; Newman and Schwager, 1993). For students who perceived high levels of environmental fixed mindset, asking for help from others could be attributed to a lack of ability (Blackwell et al., 2007; Hong et al., 1999), which they view as a threat to their self-esteem. This attribution leads students to concentrate on external indicators of success, focusing on completing tasks rather than developing a deeper understanding of the material (Dweck and Leggett, 1988). Thus, these students may prioritize expedient work completion, such as obtaining answers from others when they do not understand their tasks and need assistance.

Notably, students’ environmental fixed mindset was not associated with their avoidant help-seeking, which was a somewhat unexpected finding. We suspected that similar to performance goal structure, environmental fixed mindset may have led students to adopt performance goals. When students perceive performance goal structure, an emphasis on abilities, grades, or social comparisons, they tend to focus more on getting good grades rather than on learning and task mastery (Meece et al., 2006). Under such a circumstance, avoiding help-seeking does not help as they still need to obtain answers and complete their work. Considering the positive associations between performance goals and students’ engagement in surface learning reported in prior research (Dupeyrat and Marine, 2005; Elliot et al., 1999; Greene and Miller, 1996), students with high environmental fixed mindset could still care about completing their task but not necessarily actual learning. Therefore, environmental fixed mindset may not contribute to students’ avoidance of help-seeking. Prior research has documented the relations between perceived performance goal structure and expedient help-seeking (Karabenick, 2004; Ryan et al., 1998), yet our speculation should be examined in future research.

Sense of belonging and environmental fixed mindset and their relations to adaptive, expedient, and avoidant help-seeking lend support to the conclusion that students’ perceptions of learning contexts could inform if and how they seek help in their learning. As researchers pointed out (Karabenick and Berger, 2013; Ryan et al., 2001), students’ cognitive capabilities to monitor and reflect on their performance alone cannot explain students’ decision to ask for help or not. Academic help-seeking is a socially mediated self-regulated learning strategy in which students use other social figures as a resource to secure necessary assistance for their optimal learning. Our findings highlight the importance of academic and social contexts in which students are situated for understanding their academic help-seeking.

More broadly, our findings align with prior work indicating that sense of belonging and environmental fixed mindset could facilitate students’ engagement in self-regulated learning. Specifically, sense of belonging has been linked to students’ motivation and their use of self-regulatory strategies (e.g., Goodenow, 1993; Goodenow and Grady, 1993; Kennedy and Tuckman, 2013; Won et al., 2018), both of which are major components of self-regulated learning (Pintrich and Zusho, 2007). Our findings contribute to the literature by demonstrating the role of sense of belonging in academic help-seeking. Similarly, research has shown that individuals’ growth mindset is positively associated with adaptive patterns of motivation and self-regulatory strategies (Blackwell et al., 2007; Burnette et al., 2013; Yeager and Dweck, 2012), as it encourages students to embrace challenges and persist in the face of difficulties. This study extends this line of research by revealing that students’ perceptions of their instructors’ and peers’ mindsets could also play a role in shaping self-regulated learning.

Another noteworthy contribution of this study is to evaluate the role of sense of belonging and environmental fixed mindset within online learning contexts. Specifically, our findings are the first to link sense of belonging and environmental fixed mindset to academic help-seeking in online learning settings. A few studies have explored contextual factors predicting students’ academic help-seeking in online learning contexts. Guided by achievement goal theory (Meece et al., 2006), for instance, Er (2016) provided valuable insights into the importance of fostering mastery goal structures for students’ academic help-seeking in online learning. The present study extends this line of work by documenting that sense of belonging and environmental fixed mindset could serve as additional critical contextual factors promoting or hindering students’ strategic engagement in online learning.

Our findings also contribute to the literature on STEM education. As noted in prior work, a fixed mindset is particularly pervasive in STEM fields (Leslie et al., 2015; Lytle and Shin, 2020). There are common stereotypes, such as pursuing STEM requires innate abilities, or innate talent is necessary for success in STEM fields. As such, students in STEM fields are more likely to perceive that their instructors and classmates endorse such beliefs (i.e., environmental fixed mindset), which possibly leads them toward expedient help-seeking strategies by focusing only on copying answers and completing tasks but not understanding learning materials or task mastery. Our findings suggest that these pervasive stereotypes in STEM fields might be one factor contributing to the high dropout rate and require intervention to promote students’ retention and choice in STEM education.

### Mediating role of academic help-seeking

Consistent with our expectations, academic help-seeking served as a mediator linking sense of belonging to retention intentions, disorganization, and major declaration status. That is, students’ perceived belongingness to their Engineering community was related to greater adaptive help-seeking and lesser avoidant help-seeking, and this pattern of academic help-seeking was, in turn, associated positively with students’ retention intentions in STEM fields and successful progression in engineering programs and negatively with disorganized studying. Prior research has consistently shown that sense of belonging plays a pivotal role in college students’ choice, persistence, and academic performance (e.g., Hausmann et al., 2007; Lewis and Hodges, 2015; Pittman and Richmond, 2007). Indeed, several studies have documented the importance of sense of belonging in STEM retention (Good et al., 2012; London et al., 2011). Our findings provide insights into one possible mechanism explaining the relations between sense of belonging and STEM retention.

Academic help-seeking also significantly mediated the relations between perceived environmental fixed mindset and students’ choices of future Engineering courses. Specifically, when students perceived that their Engineering instructors and peers deemed Engineering intelligence a fixed entity that is difficult to change or develop, they were more likely to minimize the effort required to increase their understanding and task mastery by asking for answers to problems without explanation or asking others to perform the task instead. In turn, the minimized effort in course learning was further linked to minimized effort in taking future Engineering courses. Students reportedly avoided taking more Engineering courses, particularly if they were not mandatory. These findings are partially supported by prior research reporting the indirect effects of environmental fixed mindset on college students’ intent to pursue math in the future (Good et al., 2012).

In sum, our findings suggest that academic help-seeking could be one possible pathway through which sense of belonging and environmental fixed mindset promote or hinder students’ choice, retention intentions, and academic performance. More broadly, our findings also fit models of self-regulated learning well. In most models of self-regulated learning rooted in social cognitive theory, social and contextual factors are assumed to influence educational outcomes through self-regulatory processes (Pintrich and Zusho, 2007). Consistent with this theoretical assumption, several studies have provided empirical evidence showing the mediating role of self-regulatory processes. Specifically, perceived instructional practices and teacher support have been linked to students’ academic success via their use of various self-regulatory strategies (e.g., Wang and Holcombe, 2010). Our findings support and extend this mediating role of self-regulatory processes by showing that academic help-seeking could be used to understand the indirect effects of sense of belonging and environmental fixed mindset on students’ choice, retention intentions, and academic performance in STEM fields.

Our findings also offer practical implications for STEM educators. First, to encourage students to seek assistance when needed, instructors may consider focusing on cultivating students’ sense of belonging. Specifically, STEM instructors could adopt teaching practices that encourage mutual respect, caring, and fairness, which help students feel accepted and connected (Allen et al., 2018), to promote students’ academic help-seeking and ultimately their academic success. In addition, instructors could aim to enhance students’ perceptions of teacher presence, which is considered important for developing positive relationships with teachers and peers in online settings (Rapanta et al., 2020). Second, addressing STEM students’ beliefs about their peers’ and instructors’ mindset may be another lever for promoting help-seeking strategies. As noted, stereotypical beliefs that STEM ability and intelligence are innate are common in STEM fields (Lytle and Shin, 2020). Thus, STEM instructors could consider fostering environmental growth mindset by communicating growth-oriented messages and providing instructional practices that encourage students to take on challenges and embrace mistakes as part of the learning process (Yeager and Dweck, 2020).

### Limitations and future directions

Our findings and conclusions should be understood within the context of at least three limitations. First, one limitation of the present study is the correlational and cross-sectional nature of the data. Although we postulated the predictive relations and the ordering of the constructs in the path model based on theory and prior findings, the opposite direction of predictive relations remains a plausible possibility. For instance, students who had already had high levels of intentions to remain in STEM fields might have asked for assistance necessary for learning. As such, our findings need to be replicated and strengthened by future research using experimental or longitudinal data.

Second, we relied on students’ self-report to assess academic help-seeking. Although widely used in assessing diverse aspects of self-regulated learning (Wolters and Won, 2017), criticisms of using self-report instruments and concerns over their validity are not uncommon (Karabenick and Zusho, 2015). It is also noteworthy that several different methods have been developed and introduced to assess self-regulated learning, including observing and recording students’ self-regulatory behaviors in classroom settings and recording traces of students’ self-regulatory behaviors in technology-enhanced learning environments (Azevedo and Gašević, 2019). Examining the reported relations using such observation or trace data represents an obvious path for future research.

Third, it should be noted that the sample size in our study was relatively small, and as a result, we had to perform path analyses. Due to the number of parameters that need to be estimated (Kline, 2011), we were unable to conduct structural equation modeling, which has several notable advantages, such as using latent variables and accounting for measurement errors. Relatedly, participants were recruited from a single course, which may limit the generalizability of our findings. Although all Engineering major students are required to take this introductory first-year course, it is possible that students may exhibit different patterns of help-seeking in other courses. Therefore, future research with larger, more representative samples is needed to conduct a more rigorous test using structural equation modeling and to generalize our findings.

## Conclusion

Despite these limitations, the present study offers initial insights as a preliminary investigation that focuses on the role of academic help-seeking, particularly in online STEM learning. The present study provides empirical evidence suggesting that students’ academic help-seeking could play a significant role in online STEM learning. In particular, this study expands the existing knowledge by showing its potential for promoting STEM choice, retention intentions, and major declaration. Additionally, our findings suggest that students’ social membership in their Engineering community and their perceptions of the community members’ views on Engineering intelligence and ability could inform if and how students ask for help and assistance in their learning. This study also revealed a pathway through which sense of belonging and environmental fixed mindset are related to students’ choice, retention intentions, and academic performance in STEM fields. However, given the presence of non-significant results and some hypotheses that were not supported, further research is needed to strengthen these findings and explore their broader implications. Overall, our findings support the conclusion that academic help-seeking could be considered among the growing array of factors that are increasingly recognized as critical influences on students’ online learning and STEM retention, and therefore a critical area of continued research.

## Data Availability

The data presented in this article are not available due to privacy concerns and the lack of participant consent for public sharing. Further inquiries can be directed to the corresponding author.

## References

[ref1] Allen K. Kern M. L. Vella-Brodrick D. Hattie J. Waters L. (2018). What schools need to know about fostering school belonging: a meta-analysis. Educ. Psychol. Rev. 30, 1–34. doi: 10.1007/s10648-016-9389-8

[ref2] Arbreton A. J. A. (1993). When getting help is helpful: Developmental, cognitive, and motivational influences on students’ academic help-seeking. Unpublished doctoral dissertation). The University of Michigan.

[ref3] Azevedo R. Gašević D. (2019). Analyzing multimodal multichannel data about self-regulated learning with advanced learning technologies: issues and challenges. Comput. Hum. Behav. 96, 207–210. doi: 10.1016/j.chb.2019.03.025

[ref4] Ball C. Huang K. T. Cotten S. R. Rikard R. V. (2017). Pressurizing the STEM pipeline: an expectancy-value theory analysis of youths’ STEM attitudes. J. Sci. Educ. Technol. 26, 372–382. doi: 10.1007/s10956-017-9685-1

[ref6] Blackwell L. S. Trzesniewski K. H. Dweck C. S. (2007). Implicit theories of intelligence predict achievement across an adolescent transition: a longitudinal study and an intervention. Child Dev. 78, 246–263. doi: 10.1111/j.1467-8624.2007.00995.x 17328703

[ref7] Bollen K. A. Hoyle R. H. (1990). Perceived cohesion: a conceptual and empirical examination. Soc. Forces 69, 479–504. doi: 10.2307/2579670

[ref8] Broadbent J. Lodge J. (2021). Use of live chat in higher education to support self-regulated help seeking behaviours: a comparison of online and blended learner perspectives. Int. J. Educ. Technol. High. Educ. 18, 1–20. doi: 10.1186/s41239-021-00253-2 PMC8021438 34778522

[ref9] Broadbent J. Poon W. L. (2015). Self-regulated learning strategies & academic achievement in online higher education learning environments: a systematic review. Internet High. Educ. 27, 1–13. doi: 10.1016/j.iheduc.2015.04.007

[ref10] Burnette J. L. O'Boyle E. H. VanEpps E. M. Pollack J. M. Finkel E. J. (2013). Mind-sets matter: a meta-analytic review of implicit theories and self-regulation. Psychol. Bull. 139, 655–701. doi: 10.1037/a0029531, PMID: 22866678

[ref11] Butler R. (1998). Determinants of help seeking: relations between perceived reasons for classroom help-avoidance and help-seeking behaviors in an experimental context. J. Educ. Psychol. 90, 630–643. doi: 10.1037/0022-0663.90.4.630

[ref12] Cheng K. H. Liang J. C. Tsai C. C. (2013). University students’ online academic help seeking: the role of self-regulation and information commitments. Internet High. Educ. 16, 70–77. doi: 10.1016/j.iheduc.2012.02.002

[ref13] Cheong Y. F. Pajares F. Oberman P. S. (2004). Motivation and academic help-seeking in high school computer science. Comput. Sci. Educ. 14, 3–19. doi: 10.1076/csed.14.1.3.23501

[ref14] Chyr W. L. Shen P. D. Chiang Y. C. Lin J. B. Tsai C. W. (2017). Exploring the effects of online academic help-seeking and flipped learning on improving students’ learning. J. Educ. Technol. Soc. 20, 11–23.

[ref15] Collins W. Sims B. C. (2006). “Help seeking in higher education academic support services” in Help seeking in academic settings: goals, groups, and contexts. eds. Karabenick S. A. Newman R. S. (New York, NY: Routledge), 203–224.

[ref16] Credé M. Kuncel N. R. (2008). Study habits, skills, and attitudes: the third pillar supporting collegiate academic performance. Perspect. Psychol. Sci. 3, 425–453. doi: 10.1111/j.1745-6924.2008.00089.x, PMID: 26158971

[ref17] Credé M. Phillips L. A. (2011). A meta-analytic review of the motivated strategies for learning questionnaire. Learn. Individ. Differ. 21, 337–346. doi: 10.1016/j.lindif.2011.03.002

[ref18] Dabbagh N. Kitsantas A. (2004). Supporting self-regulation in student-centered web-based learning environments. Int. J. E-Learn. 3, 40–47.

[ref19] Dupeyrat C. Marine C. (2005). Implicit theories of intelligence, goal orientation, cognitive engagement, and achievement: a test of Dweck’s model with returning to school adults. Contemp. Educ. Psychol. 30, 43–59. doi: 10.1016/j.cedpsych.2004.01.007

[ref20] Dweck C. S. Leggett E. L. (1988). A social-cognitive approach to motivation and personality. Psychol. Rev. 95, 256–273. doi: 10.1037/0033-295X.95.2.256

[ref21] Elliot A. J. McGregor H. A. Gable S. (1999). Achievement goals, study strategies, and exam performance: a mediational analysis. J. Educ. Psychol. 91, 549–563. doi: 10.1037/0022-0663.91.3.549

[ref22] Er E.. (2016). Understanding and supporting college students' help-seeking behavior (Doctoral dissertation, University of Georgia). Available at: https://getd.libs.uga.edu/pdfs/er_erkan_201608_phd.pdf (Accessed April 1, 2024).

[ref23] Fong C. J. Gonzales C. Hill-Troglin Cox C. Shinn H. B. (2023). Academic help-seeking and achievement of postsecondary students: a meta-analytic investigation. J. Educ. Psychol. 115, 1–21. doi: 10.1037/edu0000725

[ref24] Glass J. L. Sassler S. Levitte Y. Michelmore K. M. (2013). What's so special about STEM? A comparison of women's retention in STEM and professional occupations. Soc. Forces 92, 723–756. doi: 10.1093/sf/sot092, PMID: 25554713 PMC4279242

[ref25] Good C. Rattan A. Dweck C. S. (2012). Why do women opt out? Sense of belonging and women's representation in mathematics. J. Pers. Soc. Psychol. 102, 700–717. doi: 10.1037/a0026659, PMID: 22288527

[ref26] Goodenow C. (1993). Classroom belonging among early adolescent students: relationships to motivation and achievement. J. Early Adolesc. 13, 21–43. doi: 10.1177/0272431693013001002

[ref27] Goodenow C. Grady K. E. (1993). The relationship of school belonging and friends' values to academic motivation among urban adolescent students. J. Exp. Educ. 62, 60–71. doi: 10.1080/00220973.1993.9943831

[ref28] Graham M. J. Frederick J. Byars-Winston A. Hunter A. B. Handelsman J. (2013). Increasing persistence of college students in STEM. Science 341, 1455–1456. doi: 10.1126/science.1240487, PMID: 24072909 PMC10167736

[ref29] Greene B. A. Miller R. B. (1996). Influences on achievement: goals, perceived ability, and cognitive engagement. Contemp. Educ. Psychol. 21, 181–192. doi: 10.1006/ceps.1996.0015 8979871

[ref30] Gummadam P. Pittman L. D. Ioffe M. (2016). School belonging, ethnic identity, and psychological adjustment among ethnic minority college students. J. Exp. Educ. 84, 289–306. doi: 10.1080/00220973.2015.1048844

[ref31] Hausmann L. R. Schofield J. W. Woods R. L. (2007). Sense of belonging as a predictor of intentions to persist among African American and white first-year college students. Res. High. Educ. 48, 803–839. doi: 10.1007/s11162-007-9052-9

[ref32] Hausmann L. R. Ye F. Schofield J. W. Woods R. L. (2009). Sense of belonging and persistence in white and African American first-year students. Res. High. Educ. 50, 649–669. doi: 10.1007/s11162-009-9137-8

[ref33] Hensley L. C. Iaconelli R. Wolters C. A. (2022). “This weird time we’re in”: how a sudden change to remote education impacted college students’ self-regulated learning. J. Res. Technol. Educ. 54, S203–S218. doi: 10.1080/15391523.2021.1916414

[ref34] Hilts A. Part R. Bernacki M. L. (2018). The roles of social influences on student competence, relatedness, achievement, and retention in STEM. Sci. Educ. 102, 744–770. doi: 10.1002/sce.21449

[ref35] Hong Y. Y. Chiu C. Dweck C. S. Lin D. M.-S. Wan W. (1999). Implicit theories, attributions, and coping: a meaning system approach. J. Pers. Soc. Psychol. 77, 588–599. doi: 10.1037/0022-3514.77.3.588

[ref36] Horowitz G. Rabin L. A. Brodale D. L. (2013). Improving student performance in organic chemistry: help seeking behaviors and prior chemistry aptitude. J. Scholar. Teach. Learn. 13, 120–133.

[ref37] Hu L. T. Bentler P. M. (1999). Cutoff criteria for fit indexes in covariance structure analysis: conventional criteria versus new alternatives. Struct. Equ. Model. 6, 1–55. doi: 10.1080/10705519909540118

[ref38] Hurtado S. Carter D. F. (1997). Effects of college transition and perceptions of the campus racial climate on Latino college students' sense of belonging. Sociol. Educ. 70, 324–345. doi: 10.2307/2673270

[ref39] Jiang Y. Rosenzweig E. Q. Gaspard H. (2018). An expectancy-value-cost approach in predicting adolescent students’ academic motivation and achievement. Contemp. Educ. Psychol. 54, 139–152. doi: 10.1016/j.cedpsych.2018.06.005

[ref40] Karabenick S. A. (2003). Seeking help in large college classes: a person-centered approach. Contemp. Educ. Psychol. 28, 37–58. doi: 10.1016/S0361-476X(02)00012-7

[ref41] Karabenick S. A. (2004). Perceived achievement goal structure and college student help seeking. J. Educ. Psychol. 96, 569–581. doi: 10.1037/0022-0663.96.3.569

[ref42] Karabenick S. A. Berger J.-L. (2013). “Help seeking as a self-regulated learning strategy” in Applications of self-regulated learning across diverse disciplines: a tribute to Barry J. Zimmerman. eds. Bembenutty H. Cleary T. J. Kitsantas A. (Charlotte, NC: IAP Information Age Publishing), 237–261.

[ref43] Karabenick S. A. Dembo M. H. (2011). Understanding and facilitating self-regulated help seeking. New Dir. Teach. Learn. 2011, 33–43. doi: 10.1002/tl.442

[ref44] Karabenick S. A. Gonida E. N. (2018). “Academic help seeking as a self-regulated learning strategy: current issues, future directions” in Schunk D. H. Greene J. A. (Eds.). Handbook of self-regulation of learning and performance. 2nd ed (London: Routledge, Taylor & Francis Group), 421–433.

[ref45] Karabenick S. A. Knapp J. R. (1991). Relationship of academic help seeking to the use of learning strategies and other instrumental achievement behavior in college students. J. Educ. Psychol. 83, 221–230. doi: 10.1037/0022-0663.83.2.221

[ref46] Karabenick S. A. Zusho A. (2015). Examining approaches to research on self-regulated learning: conceptual and methodological considerations. Metacogn. Learn. 10, 151–163. doi: 10.1007/s11409-015-9137-3

[ref47] Kennedy G. J. Tuckman B. W. (2013). An exploration into the influence of academic and social values, procrastination, and perceived school belongingness on academic performance. Soc. Psychol. Educ. 16, 435–470. doi: 10.1007/s11218-013-9220-z

[ref48] Kitsantas A. Chow A. (2007). College students’ perceived threat and preference for seeking help in traditional, distributed, and distance learning environments. Comput. Educ. 48, 383–395. doi: 10.1016/j.compedu.2005.01.008

[ref49] Kline R. B. (2011). Principles and practice of structural equation modeling. 3rd Edn. (New York, NY: Guilford Press).

[ref50] Koenig K. Schen M. Edwards M. Bao L. (2012). Addressing STEM retention through a scientific thought and methods course. J. Coll. Sci. Teach. 41, 23–29.

[ref51] Leslie S. J. Cimpian A. Meyer M. Freeland E. (2015). Expectations of brilliance underlie gender distributions across academic disciplines. Science 347, 262–265. doi: 10.1126/science.1261375, PMID: 25593183

[ref52] Lewis K. L. Hodges S. D. (2015). Expanding the concept of belonging in academic domains: development and validation of the ability uncertainty scale. Learn. Individ. Differ. 37, 197–202. doi: 10.1016/j.lindif.2014.12.002

[ref53] Liu S. H. (2017). Relationship between the factors influencing online help-seeking and self-regulated learning among Taiwanese preservice teachers. Comput. Hum. Behav. 72, 38–45. doi: 10.1016/j.chb.2017.02.034

[ref54] London B. Rosenthal L. Levy S. R. Lobel M. (2011). The influences of perceived identity compatibility and social support on women in non-traditional fields during the college transition. Basic Appl. Soc. Psychol. 33, 304–321. doi: 10.1080/01973533.2011.614166

[ref55] Lykkegaard E. Ulriksen L. (2019). In and out of the STEM pipeline–a longitudinal study of a misleading metaphor. Int. J. Sci. Educ. 41, 1600–1625. doi: 10.1080/09500693.2019.1622054

[ref56] Lytle A. Shin J. E. (2020). Incremental beliefs, STEM efficacy and STEM interest among first-year undergraduate students. J. Sci. Educ. Technol. 29, 272–281. doi: 10.1007/s10956-020-09813-z

[ref57] MacKinnon D. P. Fairchild A. J. Fritz M. Z. (2007). Mediation analysis. Annu. Rev. Psychol. 58, 593–614. doi: 10.1146/annurev.psych.58.110405.085542, PMID: 16968208 PMC2819368

[ref58] Meece J. L. Anderman E. M. Anderman L. H. (2006). Classroom goal structure, student motivation, and academic achievement. Annu. Rev. Psychol. 57, 487–503. doi: 10.1146/annurev.psych.56.091103.070258, PMID: 16318604

[ref59] Micari M. Calkins S. (2021). Is it OK to ask? The impact of instructor openness to questions on student help-seeking and academic outcomes. Act. Learn. High. Educ. 22, 143–157. doi: 10.1177/1469787419846620

[ref60] Mihlon M. A. (2010). The role of self-theories of intelligence and self-efficacy in adaptive help-seeking by college students [Unpublished doctoral dissertation]. The City University of New York.

[ref61] Miller D. I. Wai J. (2015). The bachelor’s to Ph.D. STEM pipeline no longer leaks more women than men: a 30-year analysis. Front. Psychol. 6, 1–10. doi: 10.3389/fpsyg.2015.00037 25741293 PMC4331608

[ref62] Muthén L. K. Muthén B. O. (1998–2012). Mplus user's guide. 7th Edn. Los Angeles, CA: Muthén & Muthén.

[ref63] Nelson-Le Gall S. (1985). Help-seeking behavior in learning. Rev. Res. Educ. 12, 55–90.

[ref64] Newman R. S. (2000). Social influences on the development of children's adaptive help seeking: the role of parents, teachers, and peers. Dev. Rev. 20, 350–404. doi: 10.1006/drev.1999.0502

[ref65] Newman R. S. (2002). How self-regulated learners cope with academic difficulty: the role of adaptive help seeking. Theory Pract. 41, 132–138. doi: 10.1207/s15430421tip4102_10

[ref66] Newman R. S. Schwager M. T. (1993). Students' perceptions of the teacher and classmates in relation to reported help seeking in math class. Elem. Sch. J. 94, 3–17. doi: 10.1086/461747

[ref67] Oh H. Patrick H. Kilday J. Ryan A. (2024). The need for relatedness in college engineering: a self-determination lens on academic help seeking. J. Educ. Psychol. 116, 426–447. doi: 10.1037/edu0000831

[ref68] Osterman K. F. (2000). Students' need for belonging in the school community. Rev. Educ. Res. 70, 323–367. doi: 10.3102/00346543070003323

[ref69] Ostrove J. M. Long S. M. (2007). Social class and belonging: implications for college adjustment. Rev. High. Educ. 30, 363–389. doi: 10.1353/rhe.2007.0028

[ref70] Patterson D. A. Waya S. W. Ahuna K. H. Tinnesz C. G. Vanzile-Tamsen C. (2014). Using self-regulated learning methods to increase native American college retention. J. Coll. Stud. Retent. 16, 219–237. doi: 10.2190/CS.16.2.d

[ref71] Peck L. Stefaniak J. E. Shah S. J. (2018). The correlation of self-regulation and motivation with retention and attrition in distance education. Quart. Rev. Dist. Educ. 19, 1–15.

[ref72] Perez T. Cromley J. G. Kaplan A. (2014). The role of identity development, values, and costs in college STEM retention. J. Educ. Psychol. 106, 315–329. doi: 10.1037/a0034027

[ref73] Pintrich P. Zusho A. (2007). “Student motivation and self-regulated learning in the college classroom” in The scholarship of teaching and learning in higher education: an evidence-based perspective. eds. Perry R. Smart J. (Dordrecht, Netherlands: Springer), 731–810.

[ref74] Pittman L. D. Richmond A. (2007). Academic and psychological functioning in late adolescence: the importance of school belonging. J. Exp. Educ. 75, 270–290. doi: 10.3200/JEXE.75.4.270-292

[ref75] Rapanta C. Botturi L. Goodyear P. Guàrdia L. Koole M. (2020). Online university teaching during and after the Covid-19 crisis: refocusing teacher presence and learning activity. Postdigit. Sci. Educ. 2, 923–945. doi: 10.1007/s42438-020-00155-y PMC7339092 40477148

[ref76] Rattan A. Good C. Dweck C. S. (2012). “It's ok—not everyone can be good at math”: instructors with an entity theory comfort (and demotivate) students. J. Exp. Soc. Psychol. 48, 731–737. doi: 10.1016/j.jesp.2011.12.012

[ref77] Reparaz C. Aznárez-Sanado M. Mendoza G. (2020). Self-regulation of learning and MOOC retention. Comput. Hum. Behav. 111:106423. doi: 10.1016/j.chb.2020.106423

[ref78] Roszkowski M. J. (2013). The relationship of help-seeking inclinations to traditional predictors of academic success and first-semester college GPA. J. Coll. Orient. Transit. Retent. 21, 5–32. doi: 10.24926/jcotr.v21i1.2858

[ref79] Ryan A. M. Gheen M. H. Midgley C. (1998). Why do some students avoid asking for help? An examination of the interplay among students' academic efficacy, teachers' social–emotional role, and the classroom goal structure. J. Educ. Psychol. 90, 528–535. doi: 10.1037/0022-0663.90.3.528

[ref80] Ryan A. M. Patrick H. Shim S. S. (2005). Differential profiles of students identified by their teacher as having avoidant, appropriate, or dependent help-seeking tendencies in the classroom. J. Educ. Psychol. 97, 275–285. doi: 10.1037/0022-0663.97.2.275

[ref81] Ryan A. M. Pintrich P. R. Midgley C. (2001). Avoiding seeking help in the classroom: who and why? Educ. Psychol. Rev. 13, 93–114. doi: 10.1023/A:1009013420053

[ref82] Ryan A. M. Shim S. S. (2012). Changes in help seeking from peers during early adolescence: associations with changes in achievement and perceptions of teachers. J. Educ. Psychol. 104, 1122–1134. doi: 10.1037/a0027696

[ref83] Ryan A. M. Shim S. S. Lampkins-uThando S. A. Kiefer S. M. Thompson G. N. (2009). Do gender differences in help avoidance vary by ethnicity? An examination of African American and European American students during early adolescence. Dev. Psychol. 45, 1152–1163. doi: 10.1037/a0013916, PMID: 19586185

[ref84] Ryan A. M. Shin H. (2011). Help-seeking tendencies during early adolescence: an examination of motivational correlates and consequences for achievement. Learn. Instr. 21, 247–256. doi: 10.1016/j.learninstruc.2010.07.003

[ref85] Schworm S. Gruber H. (2012). E-learning in universities: supporting help-seeking processes by instructional prompts. Br. J. Educ. Technol. 43, 272–281. doi: 10.1111/j.1467-8535.2011.01176.x

[ref86] Seymour E. Hewitt N. M. (1997). Talking about leaving: why undergraduates leave the sciences. (Boulder, CO: Westview Press).

[ref87] Shih S. S. (2007). The role of motivational characteristics in Taiwanese sixth graders’ avoidance of help seeking in the classroom. Elem. Sch. J. 107, 473–495. doi: 10.1086/518624

[ref88] Shim S. S. Kiefer S. M. Wang C. (2013). Help seeking among peers: the role of goal structure and peer climate. J. Educ. Res. 106, 290–300. doi: 10.1080/00220671.2012.692733

[ref89] Shim S. S. Rubenstein L. D. Drapeau C. W. (2016). When perfectionism is coupled with low achievement: the effects on academic engagement and help seeking in middle school. Learn. Individ. Differ. 45, 237–244. doi: 10.1016/j.lindif.2015.12.016

[ref90] Szu E. Nandagopal K. Shavelson R. J. Lopez E. J. Penn J. H. Scharberg M.. (2011). Understanding academic performance in organic chemistry. J. Chem. Educ. 88, 1238–1242. doi: 10.1021/ed900067m

[ref91] van den Hurk A. Meelissen M. van Langen A. (2019). Interventions in education to prevent STEM pipeline leakage. Int. J. Sci. Educ. 41, 150–164. doi: 10.1080/09500693.2018.1540897

[ref92] Wang M. T. Holcombe R. (2010). Adolescents’ perceptions of school environment, engagement, and academic achievement in middle school. Am. Educ. Res. J. 47, 633–662. doi: 10.3102/0002831209361209

[ref93] Williams J. MacKinnon D. P. (2008). Resampling and distribution of the product methods for testing indirect effects in complex models. Struct. Equ. Model. 15, 23–51. doi: 10.1080/10705510701758166, PMID: 20179778 PMC2825896

[ref94] Wolters C. A. (2004). Advancing achievement goal theory: using goal structures and goal orientations to predict Students' motivation, cognition, and achievement. J. Educ. Psychol. 96, 236–250. doi: 10.1037/0022-0663.96.2.236

[ref95] Wolters C. A. Won S. (2017). “Validity and the use of self-report questionnaires to assess self-regulated learning” in Handbook of self-regulation of learning and performance (New York, NY: Routledge), 307–322.

[ref96] Won S. Hensley L. C. Wolters C. A. (2021). Brief research report: sense of belonging and academic help-seeking as self-regulated learning. J. Exp. Educ. 89, 112–124. doi: 10.1080/00220973.2019.1703095

[ref97] Won S. Wolters C. A. Mueller S. A. (2018). Sense of belonging and self-regulated learning: testing achievement goals as mediators. J. Exp. Educ. 86, 402–418. doi: 10.1080/00220973.2016.1277337

[ref98] Wu F. Fan W. Arbona C. de la Rosa-Pohl D. (2020). Self-efficacy and subjective task values in relation to choice, effort, persistence, and continuation in engineering: an expectancy-value theory perspective. Eur. J. Eng. Educ. 45, 151–163. doi: 10.1080/03043797.2019.1659231

[ref99] Yeager D. S. Dweck C. S. (2012). Mindsets that promote resilience: when students believe that personal characteristics can be developed. Educ. Psychol. 47, 302–314. doi: 10.1080/00461520.2012.722805

[ref100] Yeager D. S. Dweck C. S. (2020). What can be learned from growth mindset controversies? Am. Psychol. 75, 1269–1284. doi: 10.1037/amp0000794, PMID: 33382294 PMC8299535

[ref101] Zimmerman B. J. (1989). A social cognitive view of self-regulated academic learning. J. Educ. Psychol. 81, 329–339. doi: 10.1037/0022-0663.81.3.329

[ref102] Zimmerman B. J. (1995). Self-regulation involves more than metacognition: a social cognitive perspective. Educ. Psychol. 30, 217–221. doi: 10.1207/s15326985ep3004_8

[ref103] Zimmerman B. J. (2000). “Attaining self-regulation: a social cognitive perspective” in Handbook of self-regulation. eds. Boekaerts M. Pintrich P. R. Zeidner M. (San Diego, CA: Academic Press), 13–39.

